# Wound healing of the corneal epithelium: a review

**DOI:** 10.2478/abm-2021-0026

**Published:** 2021-10-29

**Authors:** Norzana Abd Ghafar, Nahdia Afiifah Abdul Jalil, Taty Anna Kamarudin

**Affiliations:** Pusat Perubatan Universiti Kebangsaan Malaysia, 56000 Cheras, Kuala Lumpur, Malaysia; Department of Anatomy, Faculty of Medicine, Universiti Kebangsaan Malaysia, 56000 Cheras, Kuala Lumpur, Malaysia

**Keywords:** cornea, epithelium, corneal, markers, regeneration, wound healing

## Abstract

The corneal epithelium (CE) forms the outermost layer of the cornea. Despite its thickness of only 50 μm, the CE plays a key role as an initial barrier against any insults to the eye and contributes to the light refraction onto the retina required for clear vision. In the event of an injury, the cornea is equipped with many strategies contributing to competent wound healing, including angiogenic and immune privileges, and mechanotransduction. Various factors, including growth factors, keratin, cytokines, integrins, crystallins, basement membrane, and gap junction proteins are involved in CE wound healing and serve as markers in the healing process. Studies of CE wound healing are advancing rapidly in tandem with the rise of corneal bioengineering, which employs limbal epithelial stem cells as the primary source of cells utilizing various types of biomaterials as substrates.

The cornea is a transparent avascular tissue, which is vital for light refraction [1]. The corneal epithelium (CE) is located at the most anterior layer of the eye, consisting of numerous cells arranged in layers that are firmly sealed together by tight junctions. These tight junctions protect the internal structure of the eyes from noxious agents [2]. Furthermore, continuous turnover of the CE plays a key role in providing protection against insults. The limbal epithelial stem cells (LESCs) located at the corneoscleral junction are the primary source for CE replenishment [3]. The synchronization of the proliferation in the basal cell layer, differentiation of wing cells and superficial cells, and finally desquamation of the superficial cells into the tear fluid contribute to the dynamic equilibrium of corneal homeostasis and wound healing [4, 5].

In the United States, corneal abrasion accounts for approximately 14% of all emergent ocular disorders [6]. Patients with this condition usually present with severe pain, photophobia, eye tearing, and blurry vision. The defects in the CE are often caused by mechanical trauma, foreign bodies, extended contact lens wear, or chemical and flash burns. In some eye diseases, such as dry eyes and neurotrophic eyes, corneal abrasion may occur spontaneously. Infection such as trachoma causes trichiasis, which abrades the corneal surface leading to corneal opacity and eventually blindness [7]. Other pathological conditions, such as corneal hypesthesia, diabetic keratopathy, and limbal stem cell deficiency may cause a delay in corneal re-epithelization, resulting in persistent epithelial defects. In limbal stem cell deficiency, inadequate LESCs and their association with disturbance in their niche microenvironment result in an unstable ocular surface, thinning of the CE, and subsequent persistent epithelial defects [3].

Proper healing of the CE is essential to maintain corneal transparency. Nonetheless, functional and structural integration between the corneal layers also plays an important role in corneal transparency [8]. Corneal stroma, a collagen-rich central layer, makes up almost 90% of the corneal thickness, formed by precise organization of parallel fibrils packed in lamella, collagen-synthesizing keratocytes, and extracellular matrix. Keratocytes, resident cells of the corneal stroma, produce glycosaminoglycans and matrix metalloproteinases that are essential for corneal transparency [8]. Endothelium, the innermost corneal layer, is a single layer of nonmitotic cells separating the cornea from the anterior chamber of the eye. The influx of anterior chamber fluid to the cornea is constantly removed by Na^+^-K^+^-ATPase pumps, creating an equilibrium between corneal dehydration and transparency. The density of corneal endothelial cells reduces with age; therefore, endothelial cellular remodeling plays a role to compensate for this. Certain conditions such as endothelial dysfunction and keratoplasty may cause a substantial decrease in endothelial cell density, leading to corneal edema and haze [9].

This review focuses on the anatomy of the CE and processes associated with its wound healing, namely, mechanotransduction, and immune and angiogenic privileges. We also highlight the factors related to CE wound healing, including growth factors, keratin, cytokines, integrins, crystallins, basement membrane (BM), and gap junction proteins, which are essential CE wound healing markers. Corneal regeneration using various biomaterials is also discussed, as this technique has become increasingly prominent in CE wound healing studies.

## Methods

A literature search relevant to this review was conducted by searching PubMed and Scopus databases, and by analyzing the reference lists of relevant articles to identify other studies that were not found in the database searches, without limitation on years or types of publication. The search terms used were as follows: “corneal epithelium”, “wound healing”, “basement membrane”, “keratin”, “growth factor”, “cytokine”, “laminin”, “gap junction”, “perlecan”, “nidogen”, “corneal regeneration”, “crystallin”, “immune cell”, and “angiogenic”. All articles were screened before inclusion in this review.

## Corneal epithelium

**Figure 1:**
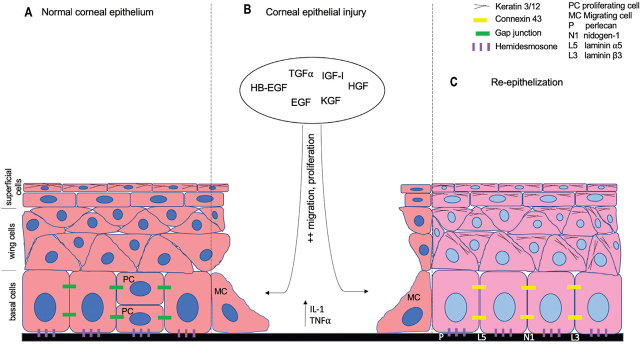
(**A**) Three main cell types that form the CE. (**B**) Various growth factors and cytokines are produced in response to injury for the wound healing process. (**C**) Few markers for nascent regenerating CE and the BM. BM, basement membrane; CE, corneal epithelium.

The cornea is an optically clear, dome-shaped structure with a steep curvature that provides approximately two-thirds of the eye’s refractive power. Microscopically, the cornea has a remarkable regularity and evenness, and consists of 3 cell layers, namely, the epithelium, stroma, and endothelium. These layers are separated by 2 membranes: the Bowman and Descemet membranes [1]. The CE is made up of 5–7 layers of nonkeratinized stratified squamous epithelium with an approximate thickness of 50 μm in humans. Three different types of epithelial cells are identified (**Figure 1A**. Superficial cells, wing cells, and columnar basal cells [1, 8]).

The superficial cells are the terminally differentiated cells, which form 2–4 layers on the corneal surface. They constitute a very smooth surface when observed under a light microscope. The apical membrane of the superficial layer has numerous microplicae and microvilli, giving the appearance of a rough and fine irregular surface. The overlying tear film provides a smooth and regular surface to the cornea, masking the fine irregularity of the CE. The tear film provides nutrients for the epithelium and is rich in antimicrobial properties [1]. The lateral domain of the cells is equipped with desmosomes, adherens junctions, and tight junctions, which seal the neighboring cells tightly together and prevent the invasion of pathogenic organisms into the internal milieu [1, 8]. The wing cells, 2–3 layers thick, overlying the columnar basal cells, are in an intermediate state of differentiation and rich in tonofilaments or keratin, being held together by tight junctional complexes [1, 8]. The basal cells form a monolayer of columnar-shaped cells, in contact with the underlying BM. They are the only corneal epithelial cells (CECs) that possess the mitotic capacity to differentiate into the wing and superficial cells. The columnar basal cells are the tallest cells and contribute to 40% of the total epithelial thickness [1]. Hemidesmosomes firmly fix the basal cells to the underlying stroma by forming anchoring plaques together with anchoring fibrils. The Bowman membrane separates the epithelium from the stroma and has a thickness of approximately 0.2 μm. It is formed mainly from collagen type IV, laminin, and heparan sulfate proteoglycans [10].

LESCs reside in highly vascularized papillae named the palisades of Vogt, a transition zone between the cornea and sclera, and are chief suppliers for corneal turnover [3]. Quantitative lineage tracing conducted by Altshuler et al. discovered a dual population of LESC in the limbus, with quiescent LESCs expressing Krt15/Gpha2/Ifitm3/Cd63 proteins residing in the outer limbus, while the inner limbus houses active LESCs coexpressing Krt15-GFP/Atf3/Mt1-2/Socs3 [11]. In a separate study, extensive characterization of LESCs by Vattulainen et al. found 3 distinct populations during the human pluripotent stem cell (hPSC)-LESC differentiation, which are: (a) ABCG2-positive ∆Np63α-negative quiescent cells, (b) ABCG2-positive ∆Np63α-positive cells, and (c) ABCG2-negative ∆Np63α-positive CK14/CK15-expressing cells [12]. The Wnt/BMP pathway plays a key role in corneal differentiation as evidenced by culturing ABCG2-positive hPSC-LESCs with Wnt/BMP signaling modifiers exhibited a higher regenerative capacity than ∆Np63α-positive cells [12].

An intact and healthy corneal nerve is essential for CEC regeneration. The corneal sensory nerve supply originates from the ophthalmic branch of the trigeminal ganglion, which infiltrates the corneal stroma from the limbus. The ramification of these fibers forms the subepithelial plexus and ends as intraepithelial corneal nerves in the CE. Nerve fibers are important for the sensation of various stimuli including thermal, mechanical, and chemical, causing the release of neurotrophins for corneal homeostasis and wound healing [8].

A large array of immune cells resides in the cornea, forming an intricate immune network of Langerhans cells, lymphocytes, mast cells, and innate lymphoid cells. This network facilitates the cornea in its adaptive immunity and immunoregulatory mechanisms [13]. As Langerhans cells are located mainly in the conjunctival epithelium and corneal limbus, they are exposed to the external environment, thereby acting as a front-line defense of the eye surface [13]. However, several studies reported an immunosuppressive role of Langerhans cells in certain conditions, for example, suppression of T cells in infection and antigen stimulation, and weakening of the immune response in contact hypersensitivity [14, 15]. Corneal mast cells make up a portion of the corneal limbus residents. They produce neurotrophin for corneal nerve and vascular endothelial growth factor (VEGF) for the growth of limbal blood vessels, respectively [16].

About 50% of corneal immune cells are CD11b-positive macrophages, located mainly in the posterior stroma and in the central and peripheral regions of the cornea [17]. Two types of macrophage populations are present in the cornea, namely, CC motif chemokine receptor–2 (CCR2)^+^ and CCR2^−^, which are important during the inflammatory stage of the healing process. CCR2^−^ macrophages are found to appear in the stroma at embryonic day 12.5 (E12.5), where CCR2^+^ can only be detected from E17.5 [18]. Type 2 innate lymphoid cells are found mainly surrounding the corneal blood vessels and play an essential role in CE healing [19]. γδ T cells are an important innate T cell subpopulation that expresses the γδ T cell receptor, and exist in the CE for corneal homeostasis and wound healing [13]. Epithelial-resident γδ T cells support epithelial surface survival by producing fibroblast growth factor (FGF)-7, FGF-8, and insulin-like growth factor (IGF), which bind to their specific receptors on the epithelial cells [20].

## Corneal epithelial wound healing

Corneal wound healing is a complex and dynamic process involving cell death, migration, proliferation, differentiation, and extracellular matrix remodeling. The dynamic equilibrium of corneal homeostasis was originally defined by the X, Y, Z hypothesis as proposed by Thoft and Friend [5]. This hypothesis explains that the constant proliferation and differentiation of actively dividing basal cells (X) together with the centripetal movement of peripheral cells (Y) replaced the constant desquamation of the superficial CE layer (Z). Recent research also found that CE regeneration and turnover originates from LESCs. Furthermore, lineage tracing of LESCs in R26R-Confetti mice revealed that some cell masses migrate in a spiral pattern toward the central cornea, while other slow-cycling limbal populations might be in a state of dormancy, resembling quiescent stem cells [21].

The cascade of the CE healing process involves 4 continuous overlapping phases: lag phase, migration, proliferation, and adhesion [1, 4]. The lag phase is the initial preparatory phase, where the cells and ocular surface reorganize for the migration of the epithelial cells at the wound edge. The damaged cells undergo apoptosis and are shed in tear film [1]. Fibronectin aids the migration phase by providing a provisional matrix over the denuded surface, where the cells can slide easily on the area [22]. Basal cells in the wounded area dissociate from hemidesmosomes, causing the distribution of the α6β4 integrin to the surrounding area forming a weaker cell–substrate junction. The α6β4 integrin typically functions to link the cytoskeletal elements to the underlying BM [4]. The basal aspect of the cell membrane of the injured area expresses integrin α5β1 and αvβ6 receptors, where actin and vinculin will bind to form focal contacts. Adherens junctions and gap junctions are also dissociated. Gradually, the intercellular junctions are dismantled, the cell–substrate junctions are weakened, and a provisional matrix is deposited for cell migration during the lag phase [1].

The migration phase ensues when the epithelial cells alongside the unwounded region flatten and migrate as an intact sheet to cover the injury. The leading cells drag the epithelial sheet to move across the provisional matrix [1]. They migrate by forming filopodia while the cytoskeletal contractile mechanisms aid the centripetal cell movement. The cells migrate at a constant rate of 0.05–0.06 mm/h and do not show any mitotic activity [1, 4]. The zonula occludens emerges behind the leading edge of the wound and restores the epithelial barrier function even before migration is complete, and is the first junction to be rebuilt. The migrating epithelium remodels the BM, secreting laminin within 24 h to attach to the cell membrane’s integrin αvβ6, concurrent with the remodeling of desmosomes, adherens junctions, and gap junctions. CE stratifies until its original thickness is reached [1].

After 24 h of corneal wounding, the mitotic activity of LESCs reaches up to a 7-fold increase. Up to 48 h, nearly 90% of the LESCs are involved in proliferation. Notably, almost 50% of the LESCs in the healthy contralateral eye re-enter the S-phase, indicating that a systemic stem cell response is activated following injury [23]. LESCs give rise to excess transient amplifying cells (TACs) with great migratory and proliferative capacity. Although TACs have the higher proliferative capacity, they have limited cell division capacity before terminal differentiation. However, LESC-TAC activation signaling is not well understood. Certain growth factors, cytokines, and chemotactic molecules may have a role in this healing signaling, such as keratocyte growth factor (KGF), ciliary neurotrophic factor, fibroblast growth factor-2 (FGF-2), and hepatocyte growth factor (HGF) [22].

CE wound healing reaches its final stage when the epithelial layer strongly adheres to the underlying substrate after the stratification process. Hemidesmosomes provide a stable attachment and facilitate the firm linkage between basal epithelial cells and the underlying BM and stroma. Lack of hemidesmosomes causes this attachment to be fragile, exposing the CE to recurrent erosions [1].

Immune cells are drawn to the wounded area after a corneal injury. Mast cells are usually activated and degranulated, which coincides with neutrophil recruitment to the damaged cornea [13]. γδ T cells release IL-17A causing neutrophil infiltration [24]. Moreover, the number of type 2 innate lymphoid cells increased, and its deficiency slows down wound healing. Further study found that corneal type 2 innate lymphoid cell responses are associated with IL-25, IL-33, and thymic stromal lymphopoietin after corneal injury. IL-33 is produced by CD64^+^CCR2^−^ corneal macrophages [19]. CCR2^+^ macrophages enhance inflammation at the beginning of the healing process. By contrast, CCR2^−^ macrophages suppress inflammation at the late stage of healing. Both macrophage populations are necessary for the healing of injured CE, and a lack of either leads to an inflammatory imbalance [19]. These immune cells are indispensable in promoting wound healing.

The importance of intact corneal innervation is indispensable in CE wound healing. The binding of substance P to neurokinin-1 receptors stimulates epithelial healing signaling, including EGF receptor (EGFR)-serine/threonine-specific protein kinases (AKT) and Sirtuin 1 (SIRT1), which in turn restores the hyperglycemia-impaired corneal sensibility, enhancing mitochondrial function, and reduces reactive oxygen species buildup [25]. In diabetic mouse cornea, vasoactive intestinal peptide (VIP) promotes the expression of nerve growth factor, ciliary neurotrophic factor, and anti-inflammatory cytokines. Furthermore, VIP reduces the expression of proinflammatory cytokines via Sonic Hedgehog, a downstream molecule of the VIP/VIP type 1 receptor signaling pathway [26]. In diabetic mouse cornea, mesencephalic astrocyte-derived neurotrophic factor (MANF) is decreased, but subconjunctival administration of MANF-specific siRNA improved CE wound healing and promoted neuron regeneration [27]. These findings consolidated the essential role of healthy corneal nerves in CE healing.

## Angiogenic privilege

To maintain its transparency, an avascular cornea is devoid of lymphatic and blood vessels, conferring the specialized characteristic of angiogenic privilege. In response to small injuries, the angiogenic privilege protects the cornea against neovascularization to ensure corneal transparency is always maintained. Endostatin, a potent antiangiogenic molecule, suppresses the binding of vascular endothelial growth factor (VEGF) to the endothelial cells via direct interaction with kinase insert domain receptor (KDR, a type IV receptor tyrosine kinase) KDR, also known as fetal liver kinase 1 (Flk-1), which acts as a VEGF receptor [28]. Thrombospondin-1 is another antiangiogenic protein that is important for corneal transparency and is expressed by the cornea. In the absence of thrombospondin-1, VEGF-C and VEGF-D accumulate over time, creating a prolymphangiogenic milieu. Lymphangiogenesis occurs as a result of an increase in monocyte chemotactic protein-1, which explains why thrombospondin-1 deficient animals have more VEGF-C-producing macrophages in their corneas [29]. Thrombospondin-1 mitigates angiogenesis by suppressing the viability and migration of endothelial cells via CD36 [30]. The presence of decoy receptors in the CE, such as VEGF receptors-1, -2, and -3, is critical to counteract proangiogenic signals [28].

## Immune privilege

Immune privilege of the eye is a unique adaptation of immunomodulatory response against inflammatory reactions to preserve clear vision. For instance, when antigens, such as alloantigens, enter the cornea, the generation of antigen-specific regulatory T cells is initiated, resulting in an immunosuppressive response against these antigens. Only a few major histocompatibility complex (MHC) II-positive antigen-presenting cells are seen in a healthy cornea, and corneal expression of MHC I molecules is lower than in other tissues. Moreover, immunomodulatory molecules including Fas ligand (CD95L) and programmed death-ligand 1, which restrict T cell proliferation and induce T cell apoptosis, are abundant in the cornea. In addition, induction of immune tolerance by anterior chamber-associated immune deviation is important to downregulate the antigen-specific effect of delayed-type hypersensitivity reactions [31]. Immune and angiogenic privilege are closely related, as pathological corneal neovascularization occurs concurrently with loss of the immune privilege [32].

## Mechanotransduction in corneal epithelial wound healing

Physical forces upon epithelial cells create changes in the mechanical environment. This results in a cascade of intra-cellular chemical signaling, known as mechanotransduction [33]. Following physical forces, proteins undergo conformational changes to create signals, alerting the mechanosensitive systems [33]. Mechanosensitive mechanisms of cell adhesion and migration are based in the nucleus, particularly in the nuclear envelope. The nuclear envelope mechanosensitive system is responsible for the integration of mechanical signals and cellular organization during adhesion and migration [33]. Adhesion exerts tensile forces via actomyosin contractility, leading to the activation of the nuclear mechanosensitive system as well as reinforcement of linker of nucleoskeleton and cytoskeleton (LINC) [34, 35]. Additionally, tensile forces produced by the actin cap during migration cause LINC reinforcement, resulting in a persistent migration [36]. CE is said to be sensitive to fluctuations in intraocular pressure (IOP). Although the general stiffness of the cornea is not altered considerably as IOP increases, the shape of the cornea may change, causing mechanical strain to the CEC [37, 38].

Hypoxia is harmful to the CE, notably during prolonged use of contact lenses. A contact lens limits oxygen permeability to the CE, by which corneal edema, epithelial keratitis, and loss of corneal transparency may occur. Following hypoxia, the CE undergoes apoptosis via activation of Polo-like kinase (Plk3) pathway and c-Jun·AP-1 transcription complex. In contrast, human limbal stem cells undergo differentiation due to the downregulation of Plk3 [39].

## Factors related to corneal epithelial wound healing

Corneal cells express growth factors, keratin, cytokines, integrins, crystallins, BM, and gap junction proteins during wound healing (**Figure 1B**). Furthermore, some of them can be identified in the regenerating CE and BM (**Figure 1C**). These factors have their respective dynamic role in regulating wound healing (**Table 1**) and can be evaluated in experimental CE wound healing either in vivo or in vitro.

**Table 1:** Factors in corneal epithelial wound healing and their functions

**Classification**	**Factor**	**Function**
Growth factors	EGF	Promotes cell migration and proliferation
		Enhances wound closure
	HGF	Enhances cell migration
		Upregulates cell proliferation
		Suppresses corneal inflammation
	KGF	Induces corneal epithelial cell proliferation
		Enhances stem cells division
	IGF-1	Has synergistic effect with substance P for epithelial cell migration and cell attachment
	IGF-2	Induces differentiation of limbal stem cells into epithelial cells
Keratin	KRT3/12 or K3/12	Differentiation marker for terminally differentiated corneal epithelium
		Structural and mechanical support of the epithelium
Cytokines	IL-1	Master regulator in the corneal epithelial wound healing process
		Accelerates wound healing
	TNF-α	Pro-inflammatory cytokines
Basement membrane proteins	Type IV collagen	Aides cell adhesion, migration and differentiation
	Laminin	Promotes corneal epithelial cells adhesion
	Perlecan	Structural support for epithelial basement membrane
	Nidogen	Provide link between components of basement membrane
Gap junction protein	Cx43	Mediates intercellular communication
Integrins	α2, α3, α6, αV, β1, and β4	Modulate the epithelial shape during migration
		Ligand for fibronectin, laminin

Cx, connexin; EGF, epidermal growth factor; HGF, hepatocyte growth factor; IGF-1, insulin growth factor-1; IGF-2, insulin growth factor-2; IL-1, interleukin-1; KGF, keratinocyte growth factor; K3/12, keratin protein 3/12; KRT3/12, keratin gene 3/12; TNF-α, tumor necrosing factor-α

## Growth factors

Following CE injury, many growth factors are secreted and activated to play specific roles in wound healing. These factors include epidermal growth factor (EGF), HGF, KGF, insulin growth factor (IGF), and transforming growth factor α (TGF-α). Crosstalk between various growth factors provides cellular communication as part of the CE healing process [22, 40].

### Epidermal growth factor

EGF is a small polypeptide with a molecular weight of 6 kDa. It was first discovered in the submandibular gland of a male mouse [41]. Three members of the EGF family have long been known to influence CE wound healing: EGF, TGF-α, and heparin-binding EGF-like growth factor (HB-EGF) [42]. These growth factors exert their effects by binding to a 170-kDa transmembrane tyrosine kinase receptor, termed EGFR. This results in cell proliferation, migration, and tissue remodeling in the skin, cornea, and epithelial cells [42].

The binding of EGF to EGFR leads to homodimerization/heterodimerization, phosphorylation of specific tyrosine residues, and recruitment of several proteins at the intracellular portion of the receptors. The phosphorylated tyrosine residues bind to a group of cytoplasmic signaling proteins, including phospholipase C, phosphatidylinositol 3-kinase, and the guanosine triphosphatase (GTPase)-activating protein Ras. Subsequently, cells are activated to undergo migration, proliferation, and differentiation [43].

In intact CE, abundant EGFR rest in the basal cell layer. EGFR accumulates at the cytoplasm in a wounded cornea, causing an elevated level of phosphorylated EGFR up to 24 h post-wounding, which is crucial during proliferation. EGFR return to their original state when the epithelial wound heals [44]. The production of reactive oxygen species (ROS) following CECs injury activates EGF that is important for the healing of CECs damage by triggering mitochondrial autophagy through transient receptor potential cation channel M2 (TRPM2) activation [45]. Thus, a lack of increase in EGF expression in recurrent corneal erosion results in delayed wound healing [46]. In an alkaline injury, recombinant human EGF promotes CECs proliferation and anti-inflammatory response [47].

TGF-α is another EGFR ligand and expressed by CECs; yet its receptor is expressed by fibroblasts [40]. TGF-α is a potent activator of CECs migration by mediating EGFR recycling, thus prolonging EGFR signaling [48]. However, excess TGF-α adversely affects corneal differentiation, causing CECs to express a conjunctival phenotype of K13, and keratocytes to change their cell fate fate to a myofibroblast phenotype [49].

HB-EGF is yet another ligand for EGFR, and binds to both erbB1 and erbB4 receptors, which subsequently leads to EGFR activation [22]. HB-EGF has a domain that binds to negatively charged glycans on the cell surface and extra-cellular matrix (ECM), and promotes prolonged activation of EGFR [50, 51]. In HB-EGF deficient mice, prolonged CE wound healing was reversed by adding HB-EGF as it enhanced cellular attachment and proliferation [52].

### Hepatocyte growth factor

HGF or scatter factor is a glycoprotein, which is synthesized mainly by mesenchymal cells and contributes to the proliferation and renewal of tissues. Its surface receptor, c-met, is expressed in epithelial cells [52]. HGF and c-met mRNAs are expressed by the CE, stroma, and endothelial cells. It plays an essential role in the epithelial wound healing process as its expression is upregulated in keratocytes and epithelial cells, following an injury to the CE [53]. HGF is synthesized by the corneal fibroblasts, promoting CE wound healing in a paracrine manner. Following an epithelial injury, HGF exerts its effect upon binding to the c-met present on the CE. The binding of HGF to c-met phosphorylates the tyrosine residues of the intracellular tyrosine kinase domain of c-met. This pathway plays a key role for cell survival, tissue protection, and regeneration, and ameliorates chronic inflammation [52]. In addition, HGF enhances CEC migration by activating p42/44 mitogen-activated protein kinase (MAPK) [54].

In an animal model, the topical application of HGF in corneal injury enhanced CE wound healing by reversing the anti-proliferative effect of the inflammation, upregulating epithelial cell proliferation, and repressing corneal inflammation. This finding could be the basis for the development of HGF-based topical eye drops to treat persistent epithelial defects [55].

### Keratinocyte growth factor

KGF belongs to the fibroblast growth factor (FGF) family and is also known as fibroblast growth factor-7 (FGF-7) [22]. KGF is expressed by fibroblasts and CECs express KGF receptors [40]. KGF provides sustained protection of CECs against apoptosis. Its ability to downregulate the p27(kip) expression is beneficial for sustaining cell survival and growth. Additionally, KGF suppresses p53 and poly (adenosine diphosphate-ribose) polymerase, which are the proteins involved in programmed cell death and inhibition of the breakdown of G1/S cell cycle progression checkpoint protein in retinoblastoma [56]. The silencing of KGF and HGF mutually affect each other, in which KGF silencing reduces HGF expression and vice versa. This will lead to the retardation of CE proliferation in ultraviolet radiation (UVR)-induced injury, corneal neovascularization, and induction of apoptosis [57].

### Insulin growth factor

Insulin-like growth factor (IGF-1) consists of 70 amino acids forming a single chain polypeptide and 3 disulfide bridges. It is a multifunctional regulatory growth factor involved in cell growth maintenance, proliferation, differentiation, and maturation, as well as regeneration [58, 59]. CECs express IGF-1 and its receptor [60]. Upon wounding, IGF-1 level is upregulated rapidly, and it enhances the expression of IGF-1 receptor in limbal cells. This induces the activation and differentiation of limbal stem cells into CE cells expressing positive *KRT12* gene. However, IGF-1 does not promote the proliferation of limbal cells per se [58].

A previous study of de-epithelized rabbit cornea demonstrated a significant synergistic effect of substance P and IGF-1 for both epithelial migration and attachment of cells to the extracellular matrix. Both substance P and IGF-1 upregulate fibronectin receptor and enhance wound healing rate. Moreover, substance P stimulates DNA synthesis in rabbit CECs [60]. Augmentation of cell migration by insulin is probably due to the regulation of the PI3k and MAPK signaling pathways. Downstream signaling of PI3k activates small Rho GTPases that induce the formation of lamellipodia. Reorganization of the actin cytoskeleton also enhances re-epithelization [60]. Expression of IGF-1 and IGF-2 receptors are upregulated after an injury. Both IGF-2 and its receptor induce the differentiation of limbal stem cells into epithelial cells [61].

## Keratin

Keratin is one of the major groups of intermediate filaments, providing structural and mechanical support of the epithelium. Differentiation of CECs can be identified via keratin expression. Corneal basal TAC, because of centripetal migration and differentiation of LESCs, coexpress K5/14 and K3/12. As these cells stratify and reach apical layers, they form suprabasal CECs that express K3/12 only [62]. K19 is considered a minor cytoskeletal component of CE, yet one of the primary cytoskeletal elements for conjunctival epithelium [62].

Terminal differentiation of CE expressing K3/12 is vital for reserving the CEC phenotype, corneal transparency, and restoring clear vision. Disturbance in the healing process could cause mixed keratin expression and impaired visual acuity [63]. Ocular surface burns treated with autologous simple limbal epithelial transplantation demonstrated regenerated CE expressing K3/12 with the absence of conjunctival cells (Muc5AC^−^/K19^−^) and focal reservation of ΔNp63α(^+^)/ABCG2(^+^) limbal stem cells [64]. Healing after successfully implanting contralateral healthy differentiated limbal-CE on patients with an eye having limbal stem cell deficiency was characterized by upregulation of K3 expression, with clinical improvement in visual acuity. By contrast, a case of failure had K19 staining with poor K3 staining [63]. In a model of corneal abrasion wound healing in vitro, the regenerated CE manifested upregulation of both K3 and KRT3 expression in CECs cultured in acacia honey-supplemented media [65]. In a wound healing model, validation of regenerated epithelium is essential to confirm that the treatment given is safe and does not alter its native characteristics, which could eventually alter the function and structure of the CE. Additionally, mutation of KRT3/12 could lead to the autosomal dominant disorder known as Meesmann corneal dystrophy, causing the formation of a cyst that usually ruptures in adulthood [62].

## Cytokines

Cytokines that are released following CE injury play a key role in driving the process of wound healing, particularly interleukin 1 (IL-1) and TNF-α (**Figure 1B**) [4, 66]. The release of the cytokines influences the expression of the EGF, KGF, HGF, and TGF [59]. In diabetic corneas, IL-1 is raised and IL-1 receptor antagonist (IL-1Ra) is suppressed, causing an imbalance in IL-1 and IL-1Ra levels as well as impaired CE wound healing [67]. TNF-α is increased in dry eye and inflammatory eye conditions. TNF-α increases not only disturb the barrier function of the CE, but also impede the migration of CECs [68, 69]. A recent study demonstrated that poor CE wound healing is a result of a transient inhibition of CE stem cells caused by IL-1β and TNF-α [70]. Furthermore, consistent treatment with IL-1 or TNF after CE debridement reduces the level of phosphorylated signal transducer and activator of transcription 3 (pSTAT3), and increases the amount of cell cycle inhibitor p16Ink4a, resulting in impaired CE healing [71].

## Basement membrane proteins

The basement membrane (BM) is described as a thin acellular layer forming a specialized ECM, which lies beneath the epithelial cells [10]. The composition of the BM is unique from one tissue to another, depending on the components forming them [10, 72]. Generally, the corneal epithelial BM is made up of 4 main components, namely collagens, laminin, heparan sulfate proteoglycans, and nidogen [72]. It changes dramatically during development and appears to be regionally heterogeneous from the central cornea to the limbus and conjunctiva [73]. BM are involved in various biological activities such as cell proliferation, differentiation, growth, and migration [10]. Through their effects on the cytoskeleton, epithelial BM affects cell polarity, cell adhesion and spreading, and cell migration [10, 72]. Epithelial BM controls the passage of epithelium-derived TGF-β and platelet-derived growth factor to stromal cells, including myofibroblast precursors. The entry of the stromal-derived growth factors, such as KGF, to the epithelium is also regulated by epithelial BM. Disruption of the BM triggers the formation of myofibroblasts as there is no barrier to limit the access of the epithelial cytokines into the stromal cells [10]. The free access of TGF-β into the stromal cells promotes the development of mature myofibroblasts and inhibits apoptosis of mature myofibroblasts and their precursors, leading to a loss of corneal transparency [10].

## Collagen

Various types of collagens form the epithelial BM. Type IV collagen is a premier element of the BM, which forms interwoven networks with other BM components that aid cell adhesion, migration, and differentiation. Type IV collagen has 6 genetically different α-chains designated α1(IV) through α6(IV). The chains are assembled, forming 3 distinct heterodimers of α1α1α2, α3α4α5, and α5α5α6 [72]. Type IV collagen is a major scaffold for the mechanically stable BM [10]. During wound healing, collagen type I and IV gradually replace the fibronectin under the regenerated epithelium as the healing progresses [22]. Collagen VII, XII, XV, XVIII, and XVII are additional types of collagen present in the corneal epithelial BM [10]. Collagen VII is an essential component in anchoring fibrils, whereas collagen XVII in hemidesmosomes acts as adhesion molecules. Collagen XV and XVIII actively participate in corneal wound healing and have been linked to the corneal avascularity [73]. Perlecan and nidogens 1/2 crosslink both collagen type IV and laminin networks within the mature normal epithelial BM, providing a stable scaffold for the structural stability of the epithelial BM [74]. Perlecan also participates in the interplays with collagen type IV, laminins, and nidogens via a specific domain. These multiple interplays of epithelial BM elements are also essential for the organization and stability of epithelial BM [75]. Interaction between the central triple-helical domain of collagen type IV with integrins α1β1 and α2β1 induces adhesion, stimulates migration, and activates proliferation of CECs [74].

## Laminin

Laminins are noncollagenous components of BM, which are present in surplus. They are heterotrimeric glycoproteins made up of 3 chains: α, β, and γ. Three laminin α-chains generally formed by epithelial are α1, α3, and α5. Laminins consisting of either α1 or α3 chains are largely limited to epithelia, whereas the α5 is mainly found in endothelial and muscle BMs. They have the ability to self-assemble and form networks that remain in close association with cells through interactions with cell surface receptors [72]. As a core of the BM, they stabilize the structure of the epithelial cells, are involved in a variety of signaling cascades, and promote cell migration through the generation of traction forces [10].

LaNTα31 proteins are a family of laminins generated from *LAMA3* by alternative splicing. They are expressed abundantly by the basal layer of the conjunctival, corneal, and limbal epithelial cells. During CE wound healing, different distribution of LaNTα31 across the cell layers has been observed throughout the process. Within 24 h post-wounding, all layers showed strong staining of LaNTα31, which gradually diminished as the wound heals. However, the staining for LaNTα31 only returned to the basal layer after 72 h. These implied that the LaNTα31 protein might affect laminin-dependent processes such as wound healing [76].

## Heparan sulfate proteoglycans

Heparan sulfate proteoglycans are proteins with a core domain that is covalently linked to long linear heparan sulfate glycosaminoglycan chains. Perlecan is a major heparan sulfate proteoglycan structure in the corneal epithelial BM [10]. BM with deficient perlecan greatly impairs the CE structure. A perlecan-deficient cornea exhibited micropthalmus, thinning of CE, decreased number of proliferative cells, and reduced CE differentiation markers (Cx43, K12, Pax6, and Notch1) [77]. Following an epithelial injury, high levels of perlecan were present in the stroma and remnants of BM. Stromal keratocytes upregulate perlecan production, contributing to the regeneration of epithelial BM [78].

## Nidogen

Nidogen is another BM component that is composed of sulfated glycoproteins. Within the corneal epithelial BM, both nidogen-1 and -2 are distributed similarly. They contain 3 globular domains split by link-like and rod-like regions [10]. Generally, nidogens serve as a link protein in the corneal epithelial BM as they have a strong affinity to collagen IV and laminins [78]. In alkali-burnt corneas of rabbits, nidogen-2 positive cells started to appear by day 2 and were mainly localized in the basal layer on day 7. By day 30, all layers of regenerated CE exhibited nidogen-2 positive cells. Throughout the healing process, activated stromal cells also secreted nidogen-2. The stromal contribution may, however, be inadequate to revitalize BM structure and function in severe alkaline wounds [79].

## Gap junction proteins

The gap junction is an intercellular channel that allows the exchange of small ions or molecules <1 kDa in size. It is made up of at least 6 subunits of interchangeable shapes of connexin, which may open and close with smooth movement upon each other. Connexin is a widely expressed protein in various human tissues, including the heart, cornea, and kidney [80]. A study by Ratkay-Traub et al. showed that wounding causes the entire CE to express Cx43, which was observed mainly at the apical layer. Clusters of Cx43 were also observed in the migrating epithelium, suggesting communication between cells in the healing process [81].

Understanding the role of Cx43 in the CE wound healing leads to various research on the treatments targeting ocular Cx43 [82, 83]. A prospective study on 5 cases of persistent epithelial defect using antisense oligonucleotides on Cx43 has shown that Cx43 was blocked temporarily by the specially designed antisense oligonucleotides, causing complete re-epithelialization of the diseased eye, despite varying severity via reduction of ocular edema and remodeling of limbal perfusion [82]. The temporary knockdown of Cx43 shortened the duration of wound closure and enhanced the stratification [83]. However, these positive findings need further clinical evaluation before becoming a treatment of nonhealing epithelial defects.

## Crystallin

The ALDH3A1 protein is highly expressed in epithelial cells and stromal keratocytes in human as well as mice cornea [84]. In ALDH3-Cre transgenic mice, ALDH3 expression on the CE is detected as early as postnatal day 9 (PND9) using X-gal staining, becoming more robust by PND12. A sharp intense staining was observed from PND12 to PND28, which coincided with eye-opening [85].

The presence of ALDH3A1 and ALDH1A1 on the CE has an important structural, metabolic, and regulatory role in the cornea. ALDH3A1 and ALDH1A1 may play a key role in UVR-induced ocular damage. Reflection of UVR energy to the ocular surface can be directly absorbed by ALDH3A1, thereby protecting the inner tissues from associated damage. Another mechanism is whereby ALDH3A1 and ALDH1A1 detoxify the aldehydes produced from UVR [84]. Transfection of cells with ALDH3A1 offered a protection on an elongated cell cycle by reducing DNA synthesis and demonstrated a high level of tolerance to cytotoxic effects. The antioxidant effect of human ALDH3A1 might act via metabolism and prolonging the cell cycle [86].

ALDH1A1 and ALDH3A1 have been proposed as structural elements of the cornea. ALDH1A1 displays a significant role in transparency and refractive properties of the rabbit cornea, indicating a structural involvement as a corneal crystallin [84, 87]. Studies in vitro showed an inverse relationship between corneal crystallin levels and light scattering in rabbit keratocytes and that transfection with ALDH3A1 reduced light scattering [84, 87].

Transketolase gene expression rises steadily as the cornea matures, culminating in a burst of activity that coincides with eye opening. High expression of transketolase in the CE is believed to result from inductive processes related to environmental conditions, whereas transcriptional mechanism in the lens is associated with the developmental process [88]. Expression of ALDHA1 and transketolase gene were down-regulated in a PAX6 knockout human CECs, implying their importance to determine the role of PAX6 in regulation of CEC terminal differentiation [89].

## Integrins

Integrins are noncovalent heterodimers of α and β subunits, and are critical for the regulation of various cellular hemostatic processes, including phagocytosis, cell migration, growth, and development [90]. Previous studies have demonstrated the expression of α2, α3, α6, αV, β1, and β4 in the human CE. β4 was seen predominantly localized in the basal cells, while β1 was spread throughout the epithelial layer [91]. Upon the binding of fibronectin to α5β1, they form focal contacts that are essential for epithelial–extracellular matrix adhesion during wound healing. Another form of integrin is α6β4, which is a primary receptor for laminin-5 and a part of the hemidesmosome of adhesion junction between epithelial cells and the BM [90]. In cell migration, integrin is important in influencing the structural organization of the cytoskeleton and delivers energy from the matrix to the cytoskeleton, leading to the alteration of the epithelial shape. The establishment of a leading and trailing edge during cell migration is mainly due to integrin engagement and synergistic effects of the activity of Src family kinases and focal adhesion kinases [90].

## Corneal epithelial regeneration

Corneal epithelial regeneration is a recent breakthrough method by which to study various corneal conditions such as limbal stem cell deficiency, corneal ulcers, and corneal healing, as CECs have the ability to regenerate. Various bio-materials have been engineered to identify the substances that mimic the corneal physiology and support ocular surface regeneration [92,93,94,95].

Amniotic membrane (AM) is one of the most researched natural biomaterials, attributed to its antiangiogenic, anti-inflammatory, and neurotrophic factors [92, 96]. Topical eyedrops preparation containing cryopreserved AM and umbilical cord cells promoted re-epithelialization and restoration of corneal surface regularity in a murine model of corneal abrasion [92]. In ocular chemical burns, both AM transplantation and topical umbilical cord serum drops reduced pain and expedited re-epithelialization due to the secretion of various growth factors, enhancing tissue regeneration [96].

Hybrid nanofibrous scaffold made up of silk and polyurethane has shown remarkable features, including interconnected pores for faster nutrient diffusion and ample mechanical strength. This hybrid scaffold could support the differentiation of conjunctiva-derived mesenchymal stem cell (CJMSCs) into CE [93]. Moreover, the addition of human stromal cell-derived factor-1α (SDF-1α) to thermosensitive chitosan-gelatin hydrogel (CHI) preserved the normal structural and functional properties of CE with enhanced reconstruction and improved local expressions of growth factors required for CE repair [94].

Scaffold for corneal tissue engineering could also be formed by fabrication of a sandwich-like hybrid mat by combination of plastic-compressed collagen gel and electrospun poly(lactic-co-glycolide) (PLGA) mats. When HCECs were cultured on this mat, the cells adhered, proliferated, maintained normal phenotype, and stratified well. This implies that this hybrid mat could be used for corneal tissue bioengineering [95].

The phenotype of cultured cells can be influenced by the mechanical properties of the substrates on which the cells grow. Upon prolonged exposure of corneal stroma to inflammation in a Notch1 mutant mouse model, excessive stromal ECM was produced. Subsequently, this caused changes in the ECM mechanical properties and activation of transcriptional coactivator Yorkie (Yki) orthologs, Yes-associated protein (YAP) and transcriptional coactivator with PDZ-binding motif (WWTR1, also known as TAZ), and β-catenin signaling pathways, leading to differentiation of regenerating CE to corneal squamous cell metaplasia [97]. Culturing of HCECs on polyacrylamide substrate of various stiffness influences apoptosis, cell migration, and actin filament structures. Medium and stiff substrates with elastic modulus of 3.2 ± 0.3 kPa and 9.2 ± 1.3 kPa, respectively, supporting faster cell migration than a compliant substrate (1.3 ± 0.2 kPa). Confocal microscopic analysis of actin filaments on the compliant substrate showed less visible actin filaments, which might impair the cells’ migratory performance. Further, more apoptotic cells were observed on compliant substrates [98].

The stiffness of biomaterial substrates in corneal regeneration is known to modulate cell behavior with regards to cell migration, proliferation, morphology, and differentiation. Polydimethylsiloxane substrates with a range of elastic moduli between 10 kPa and 105 kPa displayed the native characteristics of CECs. Cells cultured on polydimethylsiloxane exhibited a typical cobblestone appearance. In contrast to softer substrates, stiffer substances influenced cells cultured on them to express higher levels of the proliferative markers, phosphorylated extracellular signal-regulated kinase (pERK) and Ki67. Phenotypic analysis showed that cells on stiffer substrates showed upregulated *KRT3* gene expression, while KRT14 expression was higher on softer substrates. By contrast, cells seeded onto softer substrates demonstrated higher levels of intermediate filaments and focal adhesions [99]. Recently, Xu et al. suggested that CE stiffness is influenced by internal factors such as age and obesity. Increased age makes the CE stiffer, whereas softer CE was found in the diabetic obese cornea. Softer CE might be due to the diffuse distribution of a polarity protein, Crumbs3 (Crb3), and PKCζ on the apical epithelium and a decrease in fibronectin. These investigators proposed that changes in CE stiffness in diabetes and matrix molecule might influence the delayed wound healing. In a mouse model of diabetic obesity a polarity protein, Crb3, and PKCζ were distributed throughout the apical epithelium and lack of lamellipodial extension is observed. Fibronectin localization was also negligible after injury [100].

## Conclusion

As the main function of the CE is to provide protection to the inner eye structures, the CE is equipped with many factors that contribute to and facilitate healing of its wounds. Some of these factors are the by-products or residents of CE and stroma, which support the restoration of the structure and functions of the CE. Because of its regenerative capability, the CE is a good candidate for bioengineering research, particularly in understanding the complexity of its wound healing.

## References

[1] Ashby BD, Garrett Q, Willcox MDP (2014). Corneal injuries and wound healing – review of processes and therapies. Austin J Clin Ophthalmol.

[2] Leong Y-Y, Tong L (2015). Barrier function in the ocular surface: from conventional paradigms to new opportunities. Ocul Surf.

[3] Saghizadeh M, Kramerov AA, Svendsen CN, Ljubimov AV (2017). Concise review: stem cells for corneal wound healing. Stem Cells.

[4] Liu C-Y, Kao WW-Y (2015). Corneal epithelial wound healing. Prog Mol Biol Transl Sci.

[5] Thoft RA, Friend J (1983). The X, Y, Z hypothesis of corneal epithelial maintenance. Invest Opthalmol Vis Sci.

[6] Channa R, Zafar SN, Canner JK, Haring RS, Schneider EB, Friedman DS (2016). Epidemiology of eye-related emergency department visits. JAMA Ophthalmol.

[7] Ahmed F, House RJ, Feldman BH (2015). Corneal abrasions and corneal foreign bodies. Prim Care.

[8] Sridhar MS (2018). Anatomy of cornea and ocular surface. Indian J Ophthalmol.

[9] Van den Bogerd B, Dhubhghaill SN, Koppen C, Tassignon MJ, Zakaria N (2018). A review of the evidence for *in vivo* corneal endothelial regeneration. Surv Ophthalmol.

[10] Wilson SE, Torricelli AAM, Marino GK (2020). Corneal epithelial basement membrane: structure, function and regeneration. Exp Eye Res.

[11] Altshuler A, Amitai-Lange A, Tarazi N, Dey S, Strinkovsky L, Bhattacharya S (2021). Discrete limbal epithelial stem cell populations mediate corneal homeostasis and wound healing. Cell Stem Cell.

[12] Vattulainen M, Ilmarinen T, Koivusalo L, Viiri K, Hongisto H, Skottman H (2019). Modulation of Wnt/BMP pathways during corneal differentiation of hPSC maintains ABCG2-positive LSC population that demonstrates increased regenerative potential. Stem Cell Res Ther.

[13] Liu J, Li Z (2021). Resident innate immune cells in the cornea. Front Immunol.

[14] Kaplan DH, Jenison MC, Saeland S, Shlomchik WD, Shlomchik MJ (2005). Epidermal Langerhans cell-deficient mice develop enhanced contact hypersensitivity. Immunity.

[15] De Agüero MG, Vocanson M, Hacini-Rachinel F, Taillardet M, Sparwasser T, Kissenpfennig A (2012). Langerhans cells protect from allergic contact dermatitis in mice by tolerizing CD8^+^ T cells and activating Foxp3^+^ regulatory T cells. J Clin Invest.

[16] Liu J, Fu T, Song F, Xue Y, Xia C, Liu P (2015). Mast cells participate in corneal development in mice. Sci Rep.

[17] Brissette-Storkus CS, Reynolds SM, Lepisto AJ, Hendricks RL (2002). Identification of a novel macrophage population in the normal mouse corneal stroma. Invest Ophthalmol Vis Sci.

[18] Liu J, Xue Y, Dong D, Xiao C, Lin C, Wang H (2017). CCR2^−^ and CCR2^+^ corneal macrophages exhibit distinct characteristics and balance inflammatory responses after epithelial abrasion. Mucosal Immunol.

[19] Liu J, Xiao C, Wang H, Xue Y, Dong D, Lin C (2017). Local group 2 innate lymphoid cells promote corneal regeneration after epithelial abrasion. Am J Pathol.

[20] Ramirez K, Witherden DA, Havran WL (2015). All hands on DE(T) C: epithelial-resident γδ T cells respond to tissue injury. Cell Immunol.

[21] Amitai-Lange A, Altshuler A, Bubley J, Dbayat N, Tiosano B, Shalom-Feuerstein R (2015). Lineage tracing of stem and progenitor cells of the murine corneal epithelium. Stem Cells.

[22] Ljubimov AV, Saghizadeh M (2015). Progress in corneal wound healing. Prog Retin Eye Res.

[23] Sagga N, Kuffová L, Vargesson N, Erskine L, Collinson JM (2018). Limbal epithelial stem cell activity and corneal epithelial cell cycle parameters in adult and aging mice. Stem Cell Res.

[24] Li Z, Burns AR, Han L, Rumbaut RE, Smith CW (2011). IL-17 and VEGF are necessary for efficient corneal nerve regeneration. Am J Pathol.

[25] Yang L, Di G, Qi X, Qu M, Wang Y, Duan H (2014). Substance P promotes diabetic corneal epithelial wound healing through molecular mechanisms mediated via the neurokinin-1 receptor. Diabetes.

[26] Zhang Y, Gao N, Wu L, Lee PS, Me R, Dai C (2020). Role of VIP and Sonic Hedgehog signaling pathways in mediating epithelial wound healing, sensory nerve regeneration, and their defects in diabetic corneas. Diabetes.

[27] Wang X, Li W, Zhou Q, Li J, Wang X, Zhang J (2020). MANF promotes diabetic corneal epithelial wound healing and nerve regeneration by attenuating hyperglycemia-induced endoplasmic reticulum stress. Diabetes.

[28] Notara M, Lentzsch A, Coroneo M, Cursiefen C (2018). The role of limbal epithelial stem cells in regulating corneal (lymph) angiogenic privilege and the micromilieu of the limbal niche following UV Exposure. Stem Cells Int.

[29] Cursiefen C, Maruyama K, Bock F, Saban D, Sadrai Z, Lawler J (2011). Thrombospondin 1 inhibits inflammatory lymphangiogenesis by CD36 ligation on monocytes. J Exp Med.

[30] Soriano-Romaní L, García-Posadas L, López-García A, Paraoan L, Diebold Y (2015). Thrombospondin-1 induces differential response in human corneal and conjunctival epithelial cells lines under *in vitro* inflammatory and apoptotic conditions. Exp Eye Res.

[31] Benhar I, London A, Schwartz M (2012). The privileged immunity of immune privileged organs: The case of the eye. Front Immunol.

[32] Cursiefen C (2007). Immune privilege and angiogenic privilege of the cornea. Chem Immunol Allergy.

[33] Aureille J, Belaadi N, Guilluy C (2017). Mechanotransduction via the nuclear envelope: a distant reflection of the cell surface. Curr Opin Cell Biol.

[34] Guilluy C, Osborne LD, Van Landeghem L, Sharek L, Superfine R, Garcia-Mata R, Burridge K (2014). Isolated nuclei adapt to force and reveal a mechanotransduction pathway in the nucleus. Nat Cell Biol.

[35] Kim DH, Wirtz D (2015). Cytoskeletal tension induces the polarized architecture of the nucleus. Biomaterials.

[36] Wu J, Kent IA, Shekhar N, Chancellor TJ, Mendonca A, Dickinson RB, Lele TP (2014). Actomyosin pulls to advance the nucleus in a migrating tissue cell. Biophys J.

[37] Johnson CS, Mian SI, Moroi S, Epstein D, Izatt J, Afshari NA (2007). Role of corneal elasticity in damping of intraocular pressure. Invest Ophthalmol Vis Sci.

[38] Pierscionek BK, Asejczyk-Widlicka M, Schachar RA (2007). The effect of changing intraocular pressure on the corneal and scleral curvatures in the fresh porcine eye. Br J Ophthalmol.

[39] Wang L, González S, Dai W, Deng S, Lu L (2016). Effect of hypoxia-regulated polo-like kinase 3 (Plk3) on human limbal stem cell differentiation. J Biol Chem.

[40] Loureiro RR, Gomes JÁP (2019). Biological modulation of corneal epithelial wound healing. Arq Bras Oftalmol.

[41] Cohen S (1962). Isolation of a mouse submaxillary gland protein accelerating incisor eruption and eyelid opening in the new-born animal. J Biol Chem.

[42] Márquez EB, De Ortueta D, Royo SB, Martínez-Carpio PA (2011). Epidermal growth factor receptor in corneal damage: update and new insights from recent reports. Cutan Ocul Toxicol.

[43] Chen J, Zeng F, Forrester SJ, Eguchi S, Zhang M-Z, Harris RC (2016). Expression and function of the epidermal growth factor receptor in physiology and disease. Physiol Rev.

[44] Zieske JD, Takahashi H, Hutcheon AEK, Dalbone AC (2000). Activation of epidermal growth factor receptor corneal during epithelial migration. Invest Ophthalmol Vis Sci.

[45] Huo Y, Chen W, Zheng X, Zhao J, Zhang Q, Hou Y (2020). The protective effect of EGF-activated ROS in human corneal epithelial cells by inducing mitochondrial autophagy via activation TRPM2. J Cell Physiol.

[46] Candar T, Asena L, Alkayid H, Altınörs DD (2020). Galectin-3, IL-1A, IL-6, and EGF levels in corneal epithelium of patients with recurrent corneal erosion syndrome. Cornea.

[47] Guan J, Zhou L, Wang L, Li X, Pan Z (2020). Germinal peptide eye drops promote corneal wound healing and decrease inflammation after alkali injury. Exp Eye Res.

[48] McClintock JL, Ceresa BP (2010). Transforming growth factor-α enhances corneal epithelial cell migration by promoting EGFR recycling. Invest Ophthalmol Vis Sci.

[49] Zhang L, Yuan Y, Yeh L-K, Dong F, Zhang J, Okada Y (2020). Excess transforming growth factor-α changed the cell properties of corneal epithelium and stroma. Invest Ophthalmol Vis Sci.

[50] Tolino MA, Block ER, Klarlund JK (2011). Brief treatment with heparin-binding EGF-like growth factor, but not with EGF, is sufficient to accelerate epithelial wound healing. Biochim Biophys Acta.

[51] Yoshioka R, Shiraishi A, Kobayashi T, Morita S-i, Hayashi Y, Higashiyama S, Obashi Y (2010). Corneal epithelial wound healing impaired in keratinocyte-specific HB-EGF-deficient mice in vivo and in vitro. Invest Ophthalmol Vis Sci.

[52] Miyagi H, Thomasy SM, Russell P, Murphy CJ (2018). The role of hepatocyte growth factor in corneal wound healing. Exp Eye Res.

[53] Kakazu A, Sharma G, Bazan HEP (2008). Association of protein tyrosine phosphatases (PTPs)-1B with c-Met receptor and modulation of corneal epithelial wound healing. Invest Ophthalmol Vis Sci.

[54] McBain VA, Forrester JV, McCaig CD (2003). HGF, MAPK, and a small physiological electric field interact during corneal epithelial cell migration. Invest Ophthalmol Vis Sci.

[55] Omoto M, Suri K, Amouzegar A, Li M, Katikireddy KR, Mittal SK, Chauhan SK (2017). Hepatocyte growth factor suppresses inflammation and promotes epithelium repair in corneal injury. Mol Ther.

[56] Chandrasekher G, Pothula S, Maharaj G, Bazan HEP (2014). Differential effects of hepatocyte growth factor and keratinocyte growth factor on corneal epithelial cell cycle protein expression, cell survival, and growth. Mol Vis.

[57] He M, Han T, Wang Y, Wu YH, Qin WS, Du LZ, Zhao CQ (2019). Effects of HGF and KGF gene silencing on vascular endothelial growth factor and its receptors in rat ultraviolet radiation-induced corneal neovascularization. Int J Mol Med.

[58] Wang C, Peng Y, Pan S, Li L (2014). Effect of insulin-like growth factor-1 on corneal surface ultrastructure and nerve regeneration of rabbit eyes after laser in situ keratomileusis. Neurosci Lett.

[59] Yu F-SX, Yin J, Xu K, Huang J (2010). Growth factors and corneal epithelial wound healing. Brain Res Bull.

[60] Shanley LJ, McCaig CD, Forrester JV, Zhao M (2004). Insulin, not leptin, promotes in vitro cell migration to heal monolayer wounds in human corneal epithelium. Invest Ophthalmol Vis Sci.

[61] Jiang Y, Ju Z, Zhang J, Liu X, Tian J, Mu G (2015). Effects of insulin-like growth factor 2 and its receptor expressions on corneal repair. Int J Clin Exp Pathol.

[62] Kao WW-Y (2020). Keratin expression by corneal and limbal stem cells during development. Exp Eye Res.

[63] Colabelli Gisoldi RAM, Pocobelli A, Villani CM, Amato D, Pellegrini G (2010). Evaluation of molecular markers in corneal regeneration by means of autologous cultures of limbal cells and keratoplasty. Cornea.

[64] Basu S, Sureka SP, Shanbhag SS, Kethiri AR, Singh V, Sangwan VS (2016). Simple limbal epithelial transplantation: long-term clinical outcomes in 125 cases of unilateral chronic ocular surface burns. Ophthalmology.

[65] Ker-Woon C, Ghafar NA, Kien Hui C, Yusof YAM, Ngah WZW (2015). The effects of acacia honey on in vitro corneal abrasion wound healing model. BMC Cell Biol.

[66] Sotozono C, He J, Matsumoto Y, Kita M, Imanishi J, Kinoshita S (1997). Cytokine expression in the alkali-burned cornea. Curr Eye Res.

[67] Yan C, Gao N, Sun H, Yin J, Lee P, Zhou L (2016). Targeting imbalance between IL-1β and IL-1 receptor antagonist ameliorates delayed epithelium wound healing in diabetic mouse corneas. Am J Pathol.

[68] Tanaka H, Fukuda K, Ishida W, Harada Y, Sumi T, Fukushima A (2013). Rebamipide increases barrier function and attenuates TNFα-induced barrier disruption and cytokine expression in human corneal epithelial cells. Br J Ophthalmol.

[69] Okada Y, Ikeda K, Yamanaka O, Miyamoto T, Kitano A, Kao WW-Y, Saika S (2007). TNFα suppression of corneal epithelium migration. Mol Vis.

[70] Yang L, Zhang S, Duan H, Dong M, Hu X, Zhang Z (2019). Different effects of pro-inflammatory factors and hyperosmotic stress on corneal epithelial stem/progenitor cells and wound healing in mice. Stem Cells Transl Med.

[71] Wang X, Zhang S, Dong M, Li Y, Zhou Q, Yang L (2020). The proinflammatory cytokines IL-1β and TNF-α modulate corneal epithelial wound healing through p16^Ink4a^ suppressing STAT3 activity. J Cell Physiol.

[72] Torricelli AAM, Singh V, Santhiago MR, Wilson SE (2013). The corneal epithelial basement membrane: structure, function, and disease. Invest Ophthalmol Vis Sci.

[73] Kabosova A, Azar DT, Bannikov GA, Campbell KP, Durbeej M, Ghohestani RF (2007). Compositional differences between infant and adult human corneal basement membranes. Invest Ophthalmol Vis Sci.

[74] Boudko SP, Danylevych N, Hudson BG, Pedchenko VK, Mecham RP (2018). Basement membrane collagen IV: isolation of functional domains. Methods in extracellular matrix biology.

[75] Kinsella MG, Wight TN, Garg HG, Linhardt RJ, Hales CA (2005). Perlecan: an extracellular matrix heparan sulfate proteoglycan that regulates key events in vascular development and disease. Chemistry and biology of heparin and heparan sulfate.

[76] Barrera V, Troughton LD, Iorio V, Liu S, Oyewole O, Sheridan CM, Hamill KJ (2018). Differential distribution of laminin N-terminus α31 across the ocular surface: implications for corneal wound repair. Invest Ophthalmol Vis Sci.

[77] Inomata T, Ebihara N, Funaki T, Matsuda A, Watanabe Y, Ning L (2012). Perlecan-deficient mutation impairs corneal epithelial structure. Invest Ophthalmol Vis Sci.

[78] Torricelli AAM, Marino GK, Santhanam A, Wu J, Singh A, Wilson SE (2015). Epithelial basement membrane proteins perlecan and nidogen-2 are up-regulated in stromal cells after epithelial injury in human corneas. Exp Eye Res.

[79] Gallego-Muñoz P, Lorenzo-Martín E, Fernández I, Herrero-Pérez C, Martínez-García MC (2019). Nidogen-2: location and expression during corneal wound healing. Exp Eye Res.

[80] Beyer EC, Berthoud VM (2018). Gap junction gene and protein families: connexins, innexins, and pannexins. Biochim Biophys Acta Biomembr.

[81] Ratkay-Traub I, Hopp B, Bor Z, Dux L, Becker DL, Krenacs T (2001). Regeneration of rabbit cornea following excimer laser photorefractive keratectomy: a study on gap junctions, epithelial junctions and epidermal growth factor receptor expression in correlation with cell proliferation. Exp Eye Res.

[82] Ormonde S, Chou C-Y, Goold L, Petsoglou C, Al-Taie R, Sherwin T (2012). Regulation of connexin43 gap junction protein triggers vascular recovery and healing in human ocular persistent epithelial defect wounds. J Membr Biol.

[83] Elbadawy HM, Mirabelli P, Xeroudaki M, Parekh M, Bertolin M, Breda C (2016). Effect of connexin 43 inhibition by the mimetic peptide Gap27 on corneal wound healing, inflammation and neovascularization. Br J Pharmacol.

[84] Chen Y, Thompson DC, Koppaka V, Jester JV, Vasiliou V (2013). Ocular aldehyde dehydrogenases: protection against ultraviolet damage and maintenance of transparency for vision. Prog Retin Eye Res.

[85] Sunny SS, Lachova J, Dupacova N, Zitova A, Kozmik Z (2020). Generation and characterization of *Aldh3-Cre* transgenic mice as a tool for conditional gene deletion in postnatal cornea. Sci Rep.

[86] Pappa A, Brown D, Koutalos Y, DeGregori J, White C, Vasiliou V (2005). Human aldehyde dehydrogenase 3A1 inhibits proliferation and promotes survival of human corneal epithelial cells. J Biol Chem.

[87] Jester JV, Brown D, Pappa A, Vasiliou V (2012). Myofibroblast differentiation modulates keratocyte crystallin protein expression, concentration, and cellular light scattering. Invest Ophthalmol Vis Sci.

[88] Sax CM, Salamon C, Kays WT, Guo J, Yu FX, Cuthbertson RA, Piatigorsky J (1996). Transketolase is a major protein in the mouse cornea. J Biol Chem.

[89] Kitazawa K, Hikichi T, Nakamura T, Sotozono C, Kinoshita S, Masui S (2017). PAX6 regulates human corneal epithelium cell identity. Exp Eye Res.

[90] McKay TB, Schlötzer-Schrehardt U, Pal-Ghosh S, Stepp MA (2020). Integrin: basement membrane adhesion by corneal epithelial and endothelial cells. Exp Eye Res.

[91] Storm RJ, Persson BD, Skalman LN, Frängsmyr L, Lindström M, Rankin G (2017). Human adenovirus type 37 uses α_v_β_1_ and α_3_β_1_ integrins for infection of human corneal cells. J Virol.

[92] Tighe S, Moein H-R, Chua L, Cheng A, Hamrah P, Tseng SCG (2017). Topical cryopreserved amniotic membrane and umbilical cord eye drops promote re-epithelialization in a murine corneal abrasion model. Invest Ophthalmol Vis Sci.

[93] Soleimanifar F, Mortazavi Y, Nadri S, Soleimani M (2017). Conjunctiva derived mesenchymal stem cell (CJMSCs) as a potential platform for differentiation into corneal epithelial cells on bioengineered electrospun scaffolds. J Biomed Mater Res A.

[94] Tang Q, Luo C, Lu B, Fu Q, Yin H, Qin Z (2017). Thermosensitive chitosan-based hydrogels releasing stromal cell derived factor-1 alpha recruit MSC for corneal epithelium regeneration. Acta Biomater.

[95] Kong B, Sun W, Chen G, Tang S, Li M, Shao Z, Mi S (2017). Tissue-engineered cornea constructed with compressed collagen and laser-perforated electrospun mat. Sci Rep.

[96] Sharma N, Singh D, Maharana PK, Kriplani A, Velpandian T, Pandey RM, Vajpayee RB (2016). Comparison of amniotic membrane transplantation and umbilical cord serum in acute ocular chemical burns: a randomized controlled trial. Am J Ophthalmol.

[97] Nowell CS, Odermatt PD, Azzolin L, Hohnel S, Wagner EF, Fantner GE (2016). Chronic inflammation imposes aberrant cell fate in regenerating epithelia through mechanotransduction. Nat Cell Biol.

[98] Molladavoodi S, Kwon H-J, Medley J, Gorbet M (2015). Human corneal epithelial cell response to substrate stiffness. Acta Biomater.

[99] Masterton S, Ahearne M (2019). Influence of polydimethylsiloxane substrate stiffness on corneal epithelial cells. R Soc Open Sci.

[100] Xu P, Londregan A, Rich C, Trinkaus-Randall V (2020). Changes in epithelial and stromal corneal stiffness occur with age and obesity. Bioengineering (Basel).

